# Nested Attention Network for Robust Medical Image Segmentation Under Digital Watermarking

**DOI:** 10.3390/biomimetics11070475

**Published:** 2026-07-08

**Authors:** Mohammad J. M. Zedan, Ahmed A. Mohammed, Mohammed A. M. Abdullah, Ersin Elbasi, Wai Lok Woo, Mohd Asyraf Zulkifley

**Affiliations:** 1 Department of Computer and Information Engineering, Ninevah University, Mosul 41002, Iraq; mohammad.jassim@uoninevah.edu.iq (M.J.M.Z.); ahmed.mohammed@uoninevah.edu.iq (A.A.M.); mohammed.abdulmuttaleb@uoninevah.edu.iq (M.A.M.A.); 2 College of Engineering and Technology, American University of the Middle East, Egaila 54200, Kuwait; ersin.elbasi@aum.edu.kw; 3 Department of Computer and Information Sciences, Northumbria University, Newcastle upon Tyne NE7 7XA, UK; wailok.woo@northumbria.ac.uk; 4 Department of Electrical, Electronic and Systems Engineering, Universiti Kebangsaan Malaysia, Bangi 43600, Malaysia

**Keywords:** digital watermarking, deep learning, semantic segmentation, medical imaging, discrete wavelet transform, least significant bit, singular value decomposition

## Abstract

Digital watermarking is widely used to protect medical images in terms of ownership, authenticity, and traceability; however, the embedding process may introduce subtle modifications that can affect the reliability of deep-learning-based clinical analysis. Existing studies have shown that watermarking has a negligible effect on medical image classification; nevertheless, its impact on segmentation performance remains insufficiently explored. Therefore, this paper aims to investigate the effects of segmentation model enhancement on watermarked medical image analysis. In this context, three representative watermarking approaches were employed, and five baseline segmentation models, namely U-Net, ResUNet++, SegNet, FCDenseNet, and TernausNet, were evaluated on two benchmark datasets: LIDC-IDRI and BRISC. Additionally, a novel deep learning model with nested attention mechanisms was specifically designed to improve feature extraction and increase sensitivity to subtle pixel-level variations in watermarked images. Segmentation performance was assessed using five standard evaluation metrics, including mean Intersection over Union (mIoU), Dice Similarity Coefficient (DSC), and the 95th percentile Hausdorff Distance (HD95). The experimental results indicate consistently minor performance degradation across both datasets. For the BRISC dataset, the reduction in mIoU ranges from 0.15% to 0.44%, while for the LIDC-IDRI dataset, it ranges from 0.19% to 0.29% compared with the no-watermarking baseline. These findings provide quantitative insight into the compatibility of watermarking techniques for medical image protection with AI-based medical image segmentation systems, highlighting their potential for broader clinical application.

## 1. Introduction

Medical images are progressively being generated, shared and stored on interconnected healthcare environments, and the ownership, authenticity and integrity of images have become a paramount demand. Digital watermarking provides a practical solution in these environments to embed auxiliary information on medical images to use in copyright protection, traceability, and authentication [1,2]. Although these benefits exist, the process of embedding watermarks can produce artifacts to the host image, which brings up significant concerns in the field of medical artificial intelligence systems regarding whether these modifications affect the reliability of the downstream deep-learning analysis [3,4,5]. This concern has become more important with the widespread adoption of deep neural networks in medical image analysis. Classification, detection, and segmentation tasks have become common along with deep learning models in a wide range of clinical applications [6]. Segmentation, in particular, plays a critical role among these tasks as it helps in the delineation of lesions, the extraction of anatomy, treatment planning, disease monitoring, and the quantitative evaluation [7,8,9]. Therefore, when watermarked medical images are incorporated into AI-assisted workflows, it is essential to assess whether watermark-induced artefacts alter image content in ways that impact segmentation performance.

In our earlier work [10], we investigated the impact of watermarking on medical image classification. The results showed that watermarking had a minimal effect on the classification accuracy in medical images across multiple convolutional neural networks and medical datasets, and the resulting reduction was as minimal as 0% up to 0.15% at most [10]. These findings suggested that watermarking preserves the image-level discriminative information required for classification. In addition, this conclusion has been reinforced by subsequent literature. Xinyun et al. [11] drew a conclusion that aligns with our earlier work [10] as supporting evidence that medical image classification remains robust after the watermarking, and thus, to draw their conclusion that digital watermarking can seamlessly integrate with deep learning-based image classification models without significantly compromising the performance of tasks in medical imaging analysis [10,11]. However, reliability in classification does not necessarily guarantee reliability in segmentation. The classification is mainly based on the fact that high-level semantic cues are preserved to allow an image to be assigned to a category [12].

Medical image classification and segmentation represent fundamentally different levels of analysis. Segmentation, in particular, requires accurate pixel-level localisation, boundary preservation, structural continuity, and region overlap fidelity. This is particularly important in medical imaging, where even minor variations in segmented boundaries have the potential to affect quantitative results, estimation of lesion burden, and interpretation that is pertinent to clinical practice [13,14]. A watermarking scheme that is harmless to a classifier may still degrade a segmentation model by shifting boundary-level features that are invisible to the human eye but meaningful to a convolutional feature extractor. Even though the concept of digital watermarking has been widely analysed in terms of imperceptibility, robustness, and extraction fidelity, and more recently, research has begun to investigate how watermarking affects the performance of medical image classification, its impact on medical image segmentation remains insufficiently explored [15,16]. Addressing this gap constitutes the central focus of the present study, especially as segmentation-based AI systems continue to gain clinical and research importance.

Motivated by this gap, we extend our prior classification-based exploration with medical image segmentation, which is more prone to local distortions. In this work, we study how representative spatial-domain and frequency-domain watermarking algorithms impact the performance of five popular segmentation architectures, U-Net [17], ResUNet++ [18], SegNet [19], FCDNet [20], and TernausNet [21], in addition to the proposed deep learning model. This paper addresses that gap through a dual contribution. First, we conduct a controlled, systematic investigation of how Least Significant Bit (LSB), Discrete Wavelet Transform (DWT), and hybrid Discrete Wavelet Transform–Singular Value Decomposition (DWT-SVD) watermarking affect inference-time segmentation performance across multiple architectures and datasets, with all models trained exclusively on clean data so that any observed degradation is attributable solely to watermark-induced modifications. Second, we propose a lightweight encoder–decoder with hierarchical attention mechanisms, specifically designed to preserve segmentation accuracy under watermark-induced perturbations, while also demonstrating strong general performance as a compact medical image segmentation model.

The analysis is conducted on two datasets, namely: LIDC-IDRI and BRISC. In order to investigate the real effect of watermarking on segmentation inference, all the models are trained on the original non-watermarked datasets, and their performance is compared using the original and watermarked testing sets.

The main contributions of this paper are summarised as follows:

1. A dedicated investigation of the effect of watermarking on deep-learning-based medical image segmentation is presented using a controlled experimental framework.
2. Five well-established segmentation architectures are benchmarked to analyse their relative sensitivity to watermarking.
3. A customised lightweight deep-learning-based segmentation network with micro-feature attention is introduced and compared against the benchmark models, achieving superior segmentation performance.

The remainder of this work is organised as follows. Section 2 reviews related work on medical image watermarking and deep-learning-based medical segmentation. Section 3 presents the adopted watermarking methods, segmentation models, datasets, and experimental design. Section 4 reports the results and discusses the observed effects of watermarking on segmentation performance. Finally, Section 5 concludes the paper and outlines future research directions.

## 2. Related Works

The integration of advanced communication networks into the medical diagnostic sector has facilitated the sharing of clinical images across servers, which makes telemedicine and collaborative consultation between experts a standard procedure in modern healthcare. Hence, incorporating digital watermarks into medical images presents numerous challenges, including the difficulty of balancing data security with the requirements of diagnostic accuracy. This is especially true in segmentation processes where the accuracy of every pixel directly affects the diagnostic decision-making of different diseases.

The U-Net architecture and its improved versions can be considered as a primary benchmark for segmenting medical images across various modalities. Building upon this foundation, many researchers have adopted this architecture by incorporating several advanced modules, such as feature fusion strategies [22], attentional mechanisms [23] and transformer-based extensions [24]. While these enhancements often achieve enhanced segmentation results, they introduce increased computational complexity and can raise concerns related to robustness, deployment feasibility and generalizability, which must be balanced to obtain reliable and efficient segmentation systems [25].

Alongside these architectural advancements, the protection of medical data during transmission has emerged as a critical research direction. Several recent survey studies have deeply reviewed digital watermarking in deep learning medical images. These studies summarise a wide range of previously employed methods based on deep learning, hybrid, frequency domain, and spatial domain approaches [26,27,28,29,30]. Additionally, these studies also address security concerns, robustness and imperceptibility metrics, watermark embedding and extraction procedures, and potential improvements in AI-based watermarking. The findings of these studies highlight the importance of striking an equilibrium between image protection requirements and maintaining image quality, thereby preserving the performance of deep learning schemes.

However, existing studies have shown that the inclusion of watermarks in poorly designed configurations can significantly degrade the performance of deep learning models. Studies by Liu et al. [31], Wei et al. [32], Lin et al. [33], and Apostolidis et al. [34] reported degradation in the segmentation and classification performance depending on the level of distortion introduced. The experimental results obtained from these studies indicate that the sensitivity of the deep models depends strongly on the watermarking strategy and the imaging modality used, despite the high degree of visual similarity between the original and watermarked images. Accordingly, the performance of segmentation models could significantly decrease as the security aspect is prioritised without considering the second aspect related to the performance of deep learning algorithms.

Conversely, a parallel set of studies has focused on developing frameworks in which watermarks can be incorporated with accurate segmentation algorithms. The authors Bayari et al. [35] proposed a protected healthcare system for kidney stone segmentation using CT images. The CT scans are encrypted by incorporating a watermarking protocol using SVD and adaptive quantisation, which they specifically designed for Internet of Medical Things (IoMT) applications. The encoder part of the proposed U-shaped segmentation model is built on the ResNeXt-50 architecture, while the decoder is provided with attention mechanisms to enhance comprehensive feature learning. The experimental results showed an F1 score of 0.9052 and a recall score of 0.8463. Additionally, the authors Denisova et al. [36] proposed an MRI diagnostic system using a three-dimensional deep neural network applied to encoded MRI images of the brain. The experimental results of this study reported a DSC score of 87.6% on the encoded MSD database, showing that the inclusion of watermarks introduces distortions into the medical images; however, their impact on automated diagnostic procedures was minimal.

While most studies on this topic have focused on embedding a single watermark with limited reliability, especially in medical applications, the authors Amrit et al. [37] have proposed a dual watermarking mechanism based on a deep convolutional neural network and principal component analysis (PCA). The proposed framework integrates UNet3+ for segmentation and targeted watermark embedding, followed by inverse PCA reconstruction. The experimental results reported a signal-to-noise ratio score of 39.59 dB and a similarity index of 0.9812 over the Xray images.

From another research perspective, a set of studies [38,39,40,41,42] has explored hybrid frameworks combining U-Net-based image segmentation with transformer-based watermarking techniques such as DWT and SVD. These hybrid approaches have demonstrated high PSNR and SSIM scores, indicating robust image security, in addition to high segmentation performance.

Recently, hybrid watermarking techniques based on transform-domain and optimisation have been adopted in medical imaging applications to overcome the trade-off between embedding capacity, invisibility, and robustness. These methods have demonstrated high visual quality, strong compression, resistance to distortion and engineering attacks, greater payload capacity, and better extraction of invisible data [43,44,45,46]. Concurrently, comparable hybridisations are underway in the field of information retrieval, where transformer-based semantic models are integrated with metaheuristic optimisation and reinforcement learning to boost the performance of query expansion and adaptability, improving the accuracy of information retrieval over the suite of benchmark datasets [47]. Collectively, these studies reflect a broader shift towards integrated approaches that use transformer-based representations, classical signal transforms, unsupervised learning, and metaheuristic optimisation to tackle complex multi-objective issues in medical data protection and intelligent information retrieval systems.

In summary, the previously reviewed studies indicate that watermark embedding can significantly impact the performance of deep learning-based segmentation systems. While some investigations have reported a noticeable decay in segmentation performance on medical images due to image distortion caused by watermark embedding, others have achieved satisfactory segmentation performance using advanced and carefully designed embedding strategies.

Nevertheless, several research gaps remain. First, most existing studies focus primarily on the strength of embedded watermarks and the protection of medical information, rather than on segmentation architectures. Second, many investigations evaluate only a single watermark embedding approach, either spatial or frequency domain, which hinders comprehensive and systematic comparisons. Third, insufficient attention has been given to the impact of embedding strength and control factors on image segmentation performance across diverse watermarking schemes and datasets. Fourth, there is a significant focus on architectures based on U-Net and its derivatives, without considering computational efficiency or the feasibility of designing lightweight architectures that ensure satisfactory performance despite distortions caused by watermark embedding.

These limitations motivate the present work, which systematically compares several methods of watermarking, investigates the embedding strength on various medical imaging datasets, and introduces a lightweight segmentation model with high diagnostic accuracy and computational efficacy.

## 3. Materials and Methods

This section describes the methodological framework adopted to assess the impact of watermarking on medical image segmentation performance. For each dataset, 80% of the images were used as the original non-watermarked training set, while the remaining 20% were reserved for testing. The segmentation models and the proposed model were trained only on the original non-watermarked training set. During evaluation, the reserved test portion was considered under four conditions: (i) the original non-watermarked test set, (ii) the corresponding LSB-watermarked test set, (iii) the corresponding DWT-watermarked test set, and (iv) the corresponding hybrid DWT-SVD-watermarked test set. In the latter three cases, the same test images were watermarked using the three adopted watermarking methods to generate three distinct watermarked versions of the test set. This framework enables a direct comparison between segmentation performance on the original test images and on their LSB-, DWT-, and hybrid DWT-SVD-watermarked counterparts. Accordingly, any observed variation in performance can be attributed to the watermarking process itself rather than to retraining effects or changes in data distribution during learning. The following subsections describe the adopted watermarking methods, segmentation models, datasets, and evaluation metrics.

### 3.1. Watermarking Methods

In order to quantify the impact of watermarking on the medical image segmentation performance, the study employed two watermarking approaches representing the spatial and frequency domains, respectively. Particularly, the LSB-based watermarking algorithm and a DWT-based watermarking algorithm were adopted from [48,49]. The application of these commonly used two techniques enables the current research to find the difference between the effect of spatial domain and transform domain watermarking on the inference of segmentation. To maintain uniformity, the same 64 × 64 binary logo watermark was employed in all experiments.

The technique in [48] relies on the LSB principle according to which the bits of the watermark are directly embedded into the pixel values of the host image. Being a representative spatial-domain method, LSB watermarking makes local intensity changes without causing a significant change in the overall image visuality. In this paper, the LSB-based approach has been adopted to investigate the effect of direct spatial-domain watermarking on the effectiveness of deep-learning-based segmentation models. The second technique in [49] involves the use of the DWT, whereby the host image is first converted to the wavelet domain, the watermark is embedded into the wavelet coefficients chosen, and then the image is reconstructed. Four watermarks were embedded in this scheme; the first three watermarks were embedded in the 1st level of DWT decomposition (i.e., LH1, HL1 and HH1) sub-bands with scaling factor α = 0.1, while the fourth watermark was embedded in the fourth level of DWT decomposition in the LL4 sub-band with α = 0.001.

Being a frequency domain-based watermarking method, DWT-based watermarking does not manipulate the pixel values; it changes the transformed image representation. This approach was applied in the current work to learn whether transform-domain embedding has a different impact on segmentation performance than spatial-domain embedding. The rationale behind the choice of these two approaches was that they have different embedding strategies and have been widely used in the literature of watermarking. It should be emphasised that the objective of this work is not to develop a new watermarking algorithm or to evaluate watermark extraction robustness. Rather, these watermarking methods are used as experimental tools to quantify the effect of watermarking on segmentation performance by comparing results obtained from the original test images and their watermarked counterparts.

Apart from the two watermarking methods adopted, a third hybrid watermarking scheme based on the DWT and SVD was presented in this work, which represents a more advanced hybrid-domain embedding scheme. The DWT-SVD method differs from the conventional DWT method that embeds the watermark in the wavelet coefficients, by embedding the watermark into the singular values of a chosen wavelet sub-band, which are well known to be perceptually stable and resistant to common image processing operations. In this scheme, the host image is first decomposed by a single-level Haar DWT to the LL_1_, LH_1_, HL_1_, and HH_1_ sub-bands and the high-frequency diagonal sub-band HH_1_ is subjected to SVD. The watermark is generated as a 64 × 64 binary logo and is predicated with a chaotic logistic map for more security, and then it is embedded by altering the singular values of HH_1_ with a scaling factor α, followed by reconstructing the watermarked sub-band and restoring the image by performing the inverse DWT. The diagonal sub-band with high frequency was chosen for embedding in order to maintain image quality, as changes in this area are the least noticeable by the human visual system. This hybrid DWT-SVD approach was added to investigate the impact on segmentation performance due to the addition of the frequency-domain decomposition and singular-value-based embedding compared to the purely LSB and conventional DWT approaches.

Algorithm 1, Algorithm 2 and Algorithm 3 summarise the LSB, DWT, and DWT-SVD watermarking procedures adopted in this study based on the methods reported in [48] and [49], respectively.
**Algorithm 1.**   **LSB-based Watermarking Procedure****Input:** Cover image I, binary watermark W **Output:** Watermarked image $I_{w}$ **Begin** 1: **Step 1:** Read the cover image caption I 2: **Step 2:** Read the binary watermark W 3: **Step 3:**
*Optional*: Apply scrambling to W to obtain $W_{s}$; otherwise, set $W_{s}\leftarrow W$ 4: **Step 4:** Partition into non-overlapping blocks 5: **Step 5:** For each block B∈I, compute horizontal and vertical gradients 6: **Step 6:** Compute gradient magnitude and direction for each block 7: **Step 7:** Apply non-maximum suppression to retain significant edge responses 8: **Step 8:** Apply hysteresis thresholding to determine valid edge-based embedding locations 9: **Step 9:** Identify the set of embedding pixels $P_{e}$ based on the detected edge locations 10: **Step 10:** Embed watermark bits from $W_{s}$ into the least significant bits of pixels in $P_{e}$ 11: **Step 11:** Reconstruct the watermarked image $I_{w}$ 12: **Return**
$I_{w}$


**Algorithm 2. DWT-based watermarking procedure**
**Input:** Cover image I, binary watermark W, embedding strength α, Arnold transforms iterations t **Output:** Watermarked image $I_{w}$ **Begin** 1: **Step 1:** Read the cover image I 2: **Step 2:** Extract the blue channel B from I 3: **Step 3:** Read the binary watermark W 4: **Step 4:** Apply Arnold transform to W for t iterations to obtain scrambled watermark $W_{s}$ 5: **Step 5:** Apply first-level discrete wavelet transform (DWT) to B and obtain sub-bands $LL_{1},LH_{1},HL_{1},HH_{1}$ 6: **Step 6:** Embed $W_{s}$ into high-frequency sub-bands: 7: $HL_{1}^{\prime }\leftarrow HL_{1}+\alpha W_{s}$ 8: $LH_{1}^{\prime }\leftarrow LH_{1}+\alpha W_{s}$ 9: $HH_{1}^{\prime }\leftarrow HH_{1}+\alpha W_{s}$ 10: **Step 7:** Perform successive wavelet decomposition up to level 4 to obtain $LL_{4}$ 11: **Step 8:** Embed $W_{s}$ into the low-frequency sub-band using reduced strength: 12: $LL_{4}^{\prime }\leftarrow LL_{4}+\alpha W_{s}$ (e.g., α=0.001) 13: **Step 9:** Perform inverse DWT to reconstruct the modified blue channel $B^{\prime }$ 14: **Step 10:** Replace the original blue channel in I with $B^{\prime }$ 15: **Return** the final watermarked image $I_{w}$


**Algorithm 3. Hybrid DWT-SVD -based watermarking procedure**
**Input: Cover image** I**, binary watermark** W**, watermark strength factor** α**, chaotic key, logistic** **control parameter** μ **Output:** Watermarked image I_w **1: Step 1:** Read the cover image I. **2: Step 2:** Convert the cover image I to grayscale and resize it to 1024 × 1024. **3: Step 3:** Read the binary watermark W and resize it to 64 × 64. **4: Step 4:** Generate a chaotic logistic sequence from the key and μ, and then apply it to scramble the watermark W through bitwise XOR to obtain the scrambled watermark W_s. **5: Step 5:** Apply first-level DWT to the cover image I to obtain the sub-bands LL_1, LH_1, HL_1, and HH_1. **6: Step 6:** Apply SVD to the high-frequency sub-band HH_1: **7**: [U,S,V]=SVD(HH_1) **8: Step 7:** Embed the scrambled watermark W_s into the singular values of HH_1 using the embedding factor α: **9:**
S′=S+αW_s **10:**
[U_1,S_1,V_1]=SVD(S′) **11:**
HH_1′=U·S_1·Vᵀ **12: Step 8:** Perform the inverse DWT using LL_1, LH_1, HL_1, and the modified sub-band HH_1’ to reconstruct the modified image $I^{\prime }$. **13: Step 9:** Output the final watermarked image I_w.

### 3.2. Baseline Segmentation Models

Medical images are characterised by their complex textures and diverse structures and modalities, which typically require robust segmentation algorithms to analyse their context and define the boundaries of areas of interest. Furthermore, the architecture of deep learning-based segmentation models varies considerably, impacting segmentation results. Therefore, it has become necessary to apply multiple models to achieve satisfactory results, as some models are better suited to the nature of medical images than others. Based on this, five fundamental comparative models, representing key milestones of semantic segmentation, were applied. U-Net is widely adopted as a baseline for biomedical image segmentation for its effective architecture to capture contextual and spatial features. ResUNet++ builds upon this framework using residual connections, attention mechanisms, and pyramid pooling to enhance feature representation. In addition, SegNet and FCDenseNet are considered for their memory-efficient upsampling and dense connectivity, respectively, whereas TernausNet is evaluated to examine the impact of a transfer learning-based encoder. The architectural characteristics of these models are summarised in Table 1. Collectively, these rich baseline models provide a comprehensive benchmark for evaluating the segmentation technique. Through these five architectures and analysis of the performance measures of the same experimental conditions, one can identify the positive contributions of each of the models. These inputs will directly affect the design of the proposed architecture, which will be designed to accurately identify pixel-level watermark detail.

### 3.3. The Proposed Segmentation Model

To examine the specific effects of watermarking on deep learning-based medical image segmentation, an optimised and lightweight segmentation architecture with hierarchical attention mechanisms, termed the Attention Guided Feature Refinement (AGFR-Net), was developed. The AGFR-Net is specifically designed to improve feature extraction and increase sensitivity to subtle pixel-level variations in watermarked images. The proposed architecture is based on traditional segmentation models, closely resembling the U-Net, which includes both encoding and decoding components and has proven effective in medical image segmentation tasks.

During the development of the AGFR-Net, the architectural design was guided by the functional characteristics of biological visual processing systems. Specifically, the encoder aims to mimic the early visual pathways of humans that hierarchically extract low-level features and spatial patterns from visual stimuli. Meanwhile, the bottleneck attention mechanisms replicate cortical attention processes by prioritising salient and informative features while suppressing less relevant signals. Conversely, the decoder and boundary refinement modules resemble perceptual integration processes responsible for reconstructing coherent feature structures and refining the visual boundaries. Relying on this biologically inspired hierarchy, the AGFR-Net gradually transforms extracted visual information into robust semantic representations, even in the presence of watermark perturbations.

**Figure 1:**
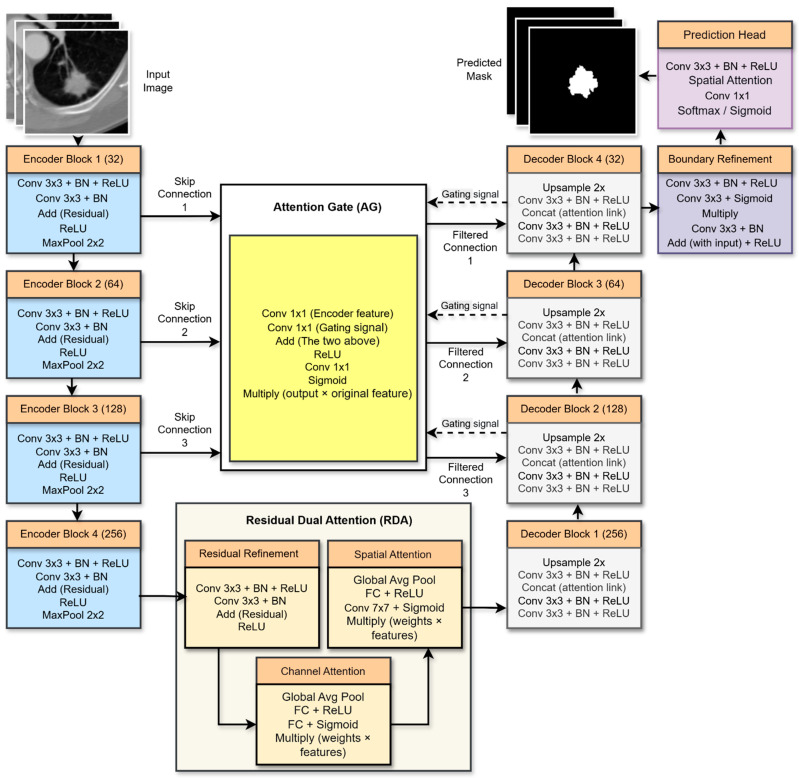
Proposed segmentation architecture AGFR-Net.

The proposed AGFR-Net (illustrated in Figure 1) primarily consists of a set of modules that work in an interconnected manner to perform fine-grained segmentation:

1. Encoder

The proposed encoder is a hierarchical feature extraction module comprising four residual convolution units that progressively extract low-level spatial features and high-level semantic information. In more detail, each encoder consists of two 3 × 3 convolutional layers, followed by a batch normalisation process and ReLU activation. The residual connections are combined to facilitate gradient flow and improve system stability. The encoder progressively increases the number of feature channels, starting with 32 across successive stages, while decreasing spatial resolution through a pooling operation.

2. Bottleneck

In the bottleneck of the proposed model, a Residual Dual Attention module (RDA) module was included to improve the quality of deep feature representations by incorporating residual feature refinement and attention mechanisms to enhance informative features and suppress irrelevant ones. Initially, the resulting features from the encoder are further refined by applying a lightweight residual convolutional block consisting of two sets of 3 × 3 convolutional layers, each followed by a batch normalisation process for the batches and a specific activation function after the first set only. The resulting feature map from the second convolution was then added to the original input feature map and activated again, ensuring further abstraction of high-level semantic features without introducing degradation or vanishing gradients and keeping the same spatial dimensions and channel depth as the input. Subsequently, a channel attention mechanism is applied to model the correlations between feature channels and enhance focus on channels containing important structural information. The refined feature maps are passed through global average pooling to produce a channel descriptor vector, which is then processed by two subsequent fully connected modules to capture nonlinearity: the first reduces the dimensionality by a reduction ratio of 16, followed by an activation process, while the second restores the original channel dimension and is activated to produce attention weights. The final output is generated by multiplying each channel by its corresponding weight to focus on the most semantically meaningful features. After channel optimisation, a spatial attention mechanism is used to highlight important spatial regions in the feature maps. The process starts with applying average pooling to capture the overall presence of features and max pooling to highlight the most salient activations, and then the resulting two maps are combined and passed through a 7 × 7 convolutional layer with a single output channel. Subsequently, the final output is activated to assign a weight for each spatial location, producing a spatial attention map that helps the network detect subtle variations that may result from watermark inclusion.

3. Skip Connections

The skip connections are also developed to connect the encoder and decoder, transmitting the encoder’s features directly to the decoder to recover spatial details. However, these transmitted features often contain irrelevant or distorted information. To address this issue, specific skip connections incorporating a lightweight module called an attention gate (AG) are proposed. This gate acts as a connection map that selectively identifies the encoder features that should be transmitted to the decoder and suppresses background noise. Each AG module accepts two inputs: the encoder feature map from a specific level of the encoder and the gating signal from the corresponding decoder stage. These two inputs are transferred, combined and then activated to produce attention coefficients between 0 and 1, where a value close to 1 indicates highly relevant information; the opposite is true for a value close to 0. This process contributes to more precise segmentation boundaries for medical images containing embedded watermarks.

4. Decoder

Conversely, a decoding side was developed to sequentially reconstruct the refined deep features from the bottleneck to produce accurate segmentation predictions for regions of interest. Each decoder block is designed with a specific fixed sequence of operations. The first operation is the upsampling that is implemented by applying the bilinear interpolation, followed by a convolutional layer. The upsampled features are then passed through a set of 3 × 3 convolutions, normalisations, and activations. The resulting refined upsampled feature maps are used to produce the gating signal that processes the corresponding encoder features. Meanwhile, the filtered encoder feature map is passed to the specific decoder stage and synergistically concatenated with the upsampled decoder feature along the channel dimension. After that, the resulting feature map, which includes rich semantic and spatial information, is passed through two consecutive 3 × 3 convolutional layers, each followed by normalisation and activation processes for more refinements and to improve segmentation representation. At this stage, the final output of the decoder consists of a full-resolution feature map of 32 channels, which may contain blurry or slightly inaccurate boundaries despite the robustness of the proposed AGFR-Net model.

5. Boundary Refinement

The resulting feature maps from the decoder are passed to the boundary refinement (BR) module. This module is located between the final decoding and segmentation modules to enhance the determination of boundaries for medically relevant areas after the decoding phase. The BR module is achieved through the application of an additional set of convolution operations that prepares the features for edge extraction. This is followed by a special edge-aware attention mechanism through another 3 × 3 convolutional layer that produces a single-channel output. This output is activated to give higher weight to structural boundaries in feature maps. After that, an element-wise multiplication is applied to selectively amplify feature responses. The boundary-enhanced features are passed through another set of 3 × 3 convolutions to refine any discontinuities introduced by the multiplication. Subsequently, an addition operation is performed between the residual connection and the output of the decoder, followed by a ReLU activation to significantly sharpen boundary representations.

6. Prediction Head

The final stage in AGFR-Net design involves the prediction head, which transforms the resulting enhanced feature maps into pixel-level predictions through a convolutional layer with 32 filters followed by a projection layer that compress the feature map along the channel dimension by applying average pooling and max pooling, where the resulting two 2D maps are concatenated and passed through a 7 × 7 convolutional layer with a unified channel, and then a sigmoid activation is applied to produce the final attention map, which is multiplied with the input feature map to produce the prediction map. These maps are then passed through a 1 × 1 convolutional layer to project the 32-channel feature representation onto the two desired output classes using an activation function that transforms initial logarithmic values into interpretable probabilities, enabling the network to detect subtle changes resulting from watermark inclusion, and at this stage it became necessary to summarize the architectural characteristics of this proposed model, as listed in Table 2, in a similar manner to what was presented in Table 1.

When comparing the details listed in Table 1 and Table 2, it can be directly inferred that the proposed network is constructed on a custom architecture that incorporates multi-scale capability and different attention methods. Moreover, the AGFR-Net features a compact design with significantly fewer parameters and reduced floating-point operations (FLOPs) compared to baseline models. From a runtime efficiency perspective, the model achieves a low inference time per image on an NVIDIA Tesla P100 GPU that translates to a high frames per second (FPS) rate. This efficiency enables the architecture to be easily deployed on resource-constrained devices and also attain a high performance in segmentation.

### 3.4. Dataset

**Figure 2:**
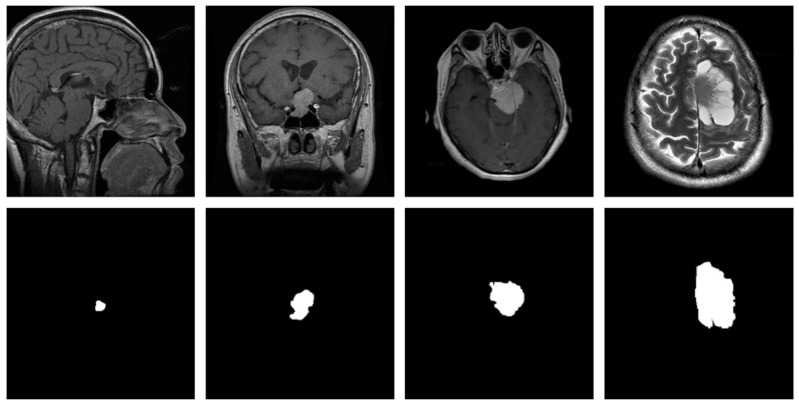
Sample images from the BRISC dataset along with their corresponding ground truth segmentation masks.

To investigate the effect of watermarking on the segmentation performance, two publicly accessible collections of medical samples with different modalities were targeted. The first batch is made up of brain MRI images called BRISC. This dataset is systematically annotated and validated by a group of experts to ensure clinical accuracy [50]. In addition, the 4793-sample BRISC dataset supports pixel-level segmentation by providing two-colour masks and also supports tumour classification for each image. Figure 2 shows some sample images and their masks from the BRISC dataset.

**Figure 3:**
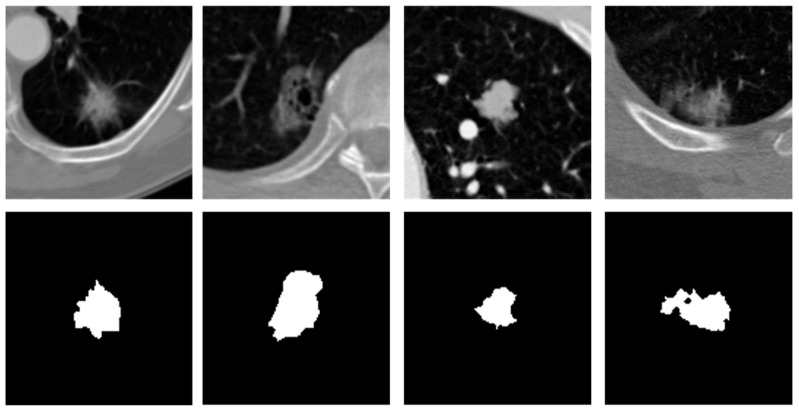
Sample images from the LIDC-IDRI dataset along with their corresponding ground truth segmentation masks.

The second segmentation-based dataset includes 14,131 samples of a 3D CT scan dataset of lung nodules from the LIDC-IDRI project. This dataset was also annotated by a panel of radiologists to capture inter-observer heterogeneity in nodule delineation. A subset of representative samples and their ground truth are illustrated in Figure 3.

These two distinct datasets were chosen to test the effect of incorporating a digital watermark into each of them while applying robust segmentation algorithms.

### 3.5. Evaluation Metrics

To comprehensively evaluate the effect of watermarking on medical image segmentation performance, five quantitative metrics were employed: overlap-based measures (mIoU and DSC), class-based measures (specificity and sensitivity), and a boundary-based measure (HD95). These metrics were selected to provide a complementary assessment of region overlap, pixel-wise classification performance, and boundary agreement between the predicted and ground-truth masks. Let TP, TN, FP, and FN denote the numbers of true positive, true negative, false positive, and false negative pixels, respectively. Based on these quantities, the adopted metrics are defined as follows:

(1) $$IoU=\frac{TP}{TP+FP+FN}$$

(2) $$mIoU=\frac{1}{N}\sum_{i=1}^{N}IoU_{i}$$

(3) $$DSC=\frac{2TP}{2TP+FP+FN}$$

(4) $$Sens.=\frac{TP}{TP+FN}$$

(5) $$Spec.=\frac{TN}{TN+FP}$$

Here, N denotes the total number of evaluated samples, and $\;IoU_{i}$ represents the Intersection over Union obtained for the i-th sample. In addition to the above overlap- and pixel-based measures, the (HD95) was used to evaluate the boundary agreement between the predicted segmentation and the ground-truth mask.

(6) $$HD95=\max(P_{95}\left(d\left(A,B\right)\right),P_{95}(d\left(B,A\right))$$

where $d\left(A,B\right)$ represents the set of the shortest distance from each point in A to the closed point of B [51]. Unlike mIoU and DSC, which quantify region overlap, HD95 determines the 95th percentile of the collection of all border point shortest distances. This is particularly relevant in medical image segmentation, where small deviations along lesion or anatomical boundaries may affect subsequent quantitative analysis and clinically relevant interpretation. By analysing the results of these adopted metrics in this study, it is easy to gain an understanding of the impact of incorporating watermarking in different methods. Specifically, the HD95 metric determines boundary accuracy, while the mIoU and the DSC measure overall overlap accuracy, and the sensitivity and specificity represent prediction behaviour at the pixel level. As a result, the adopted evaluation metrics allow for a thorough examination to determine whether the watermark negatively impacts segmentation performance at the regional and border levels.

### 3.6. Implementation Details

In this work, the suggested segmentation model AGFR-Net was constructed utilising the TensorFlow/Keras environment and trained on a cloud-based Kaggle platform using high computational distributed GPUs. Initially, the input samples are uniformly scaled and normalised. After that, a specific set of data augmentation procedures, including horizontal flipping, scaling, translation, rotation, and intensity variations, was applied for the generalisation purpose.

These processed samples are passed to the AGFR-Net architecture that was systematically trained using the grid search strategy. The search space comprises the Adam optimiser with an initial learning rate of (1 × 10^−3^, 1 × 10^−4^, 1 × 10^−5^) supported by an exponential decay rate of different dropping factors, a batch size of (4, 8, 16, 32), and a modified loss function combining Focal Tversky loss and Weighted BCE with different weighting ratios (0.5:0.5, 0.6:0.4, 0.7:0.3). The final experimental setup was designated based on the highest mIoU results on the validation set. To provide more detail on the experimental settings, the training hyperparameters used for both datasets across all models, including the proposed model, are summarised in Table 3.

## 4. Experimental Results

In this study, two reference datasets, BRISC and LIDC-IDRI, were employed to evaluate the effect of watermark on medical image segmentation. Watermarking samples were then embedded in these databases using three different methods: DWT, LSB, and hybrid DWT-SVD. Subsequently, intensive and systematic experiments were conducted using five commonly used segmentation models in addition to the proposed AGFR-Net architecture. All models were trained under identical experimental conditions to ensure transparency and impartiality, and their performance was evaluated using five standard segmentation metrics.

### 4.1. Watermarked Datasets

#### 4.1.1. DWT-Based Watermarking Results

**Figure 4:**
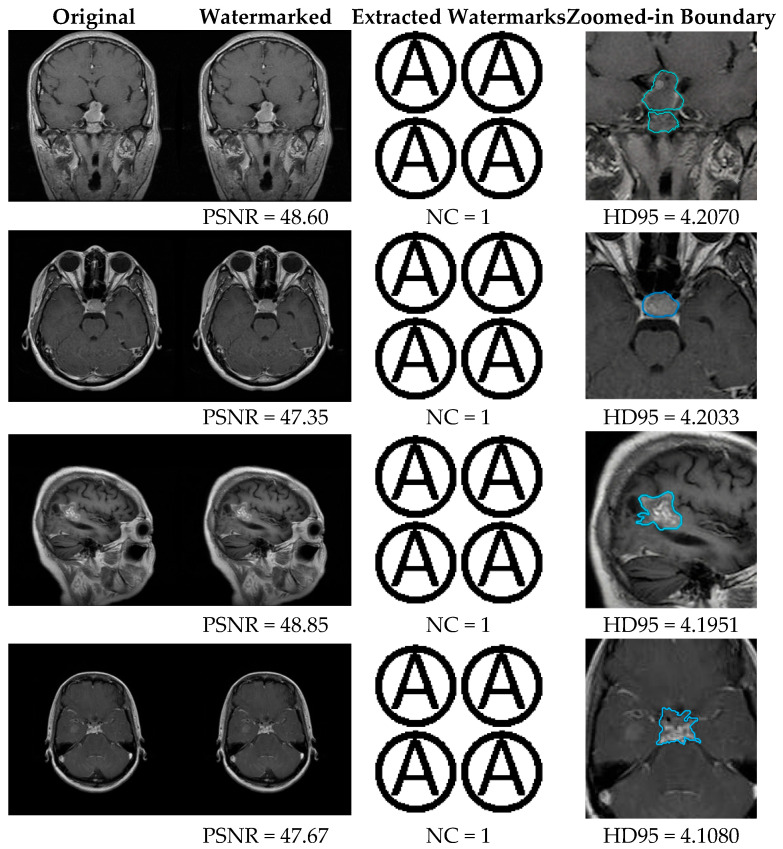
BRISC Dataset original vs. DWT-based watermarked samples, images and their watermarks extractions, and zoomed-in boundary overlay visualisations with α_1_ = 0.001 and α_2_ = 0.1.

For the BRISC Dataset, Figure 4 illustrates the imperceptibility and extracted watermarks of the DWT-based watermarking scheme.

**Figure 5:**
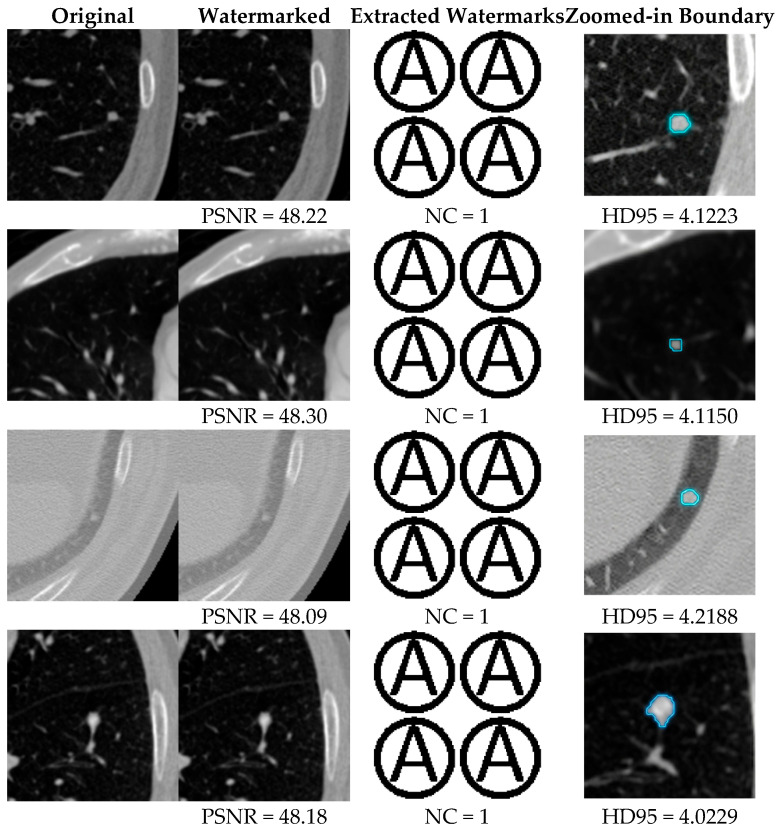
LIDC-IDRI Dataset original vs. DWT-based watermarked samples images and their watermark extractions, and zoomed-in boundary overlay visualisations with *α*_1_ = 0.001 and *α*_2_ = 0.1.

For the LIDC-IDRI Dataset, Figure 5 illustrates the imperceptibility of the DWT-based watermarking scheme.

The visual comparison shows that the watermarked images are very similar to the original ones, and no distortion of the key anatomical structures is evident. In both watermarking methods, the reported PSNR values range from 47 to 49 dB across both datasets, which confirms that there is no high visual distortion. Moreover, the extracted watermarks obtained an NC value of 1 under no-attack conditions for all evaluated samples, which indicates a perfect similarity between the extracted and original watermarks. In general, the DWT-based watermarking approach maintained a high image fidelity in both datasets.

#### 4.1.2. LSB-Based Watermarking Results

**Figure 6:**
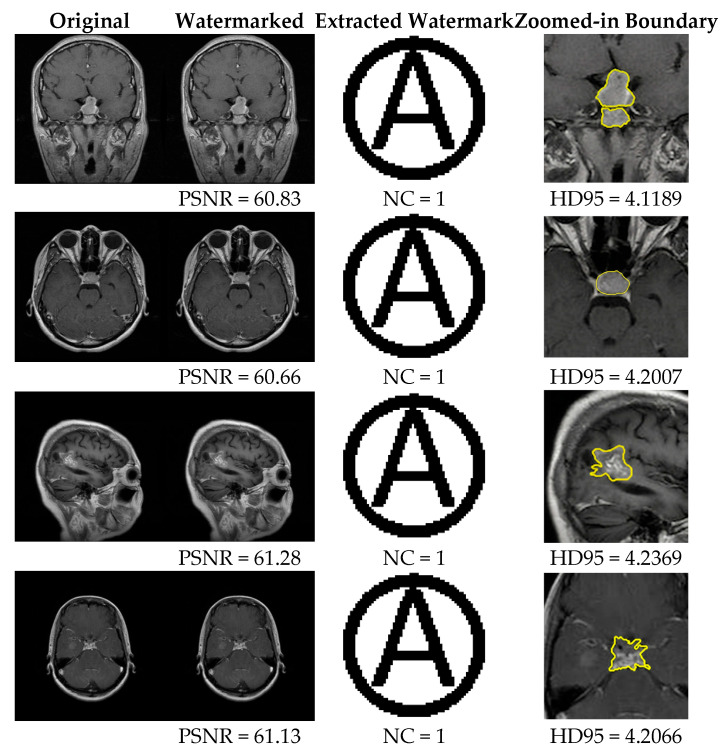
BRISC Dataset original vs. LSB-based watermarked samples images, and zoomed-in boundary overlay visualisations.

**Figure 7:**
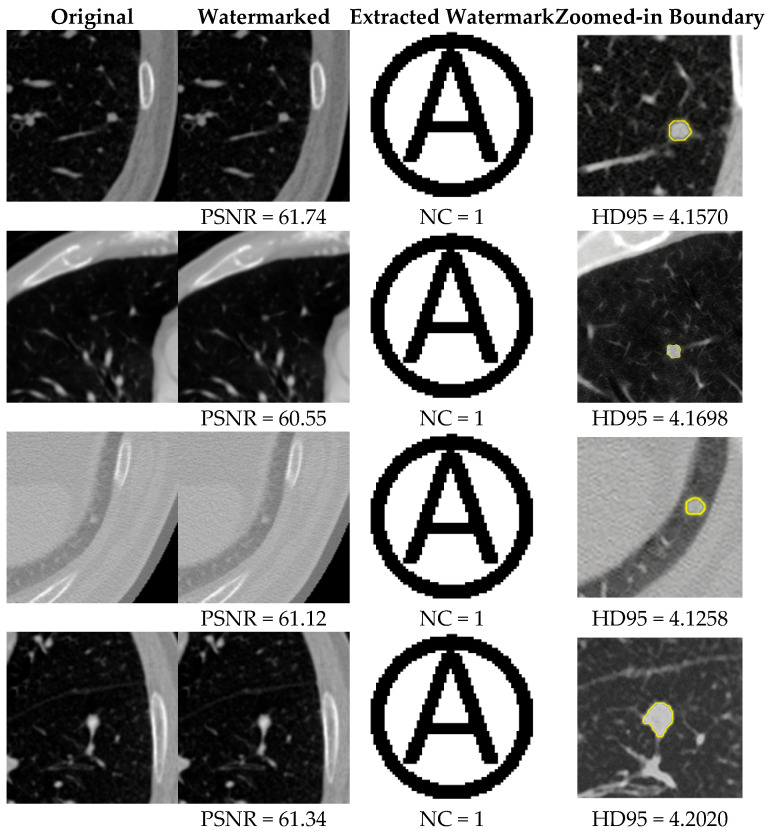
LIDC-IDRI Dataset original vs. LSB-based watermarked samples, images and zoomed-in boundary overlay visualisations.

Figure 6 and Figure 7 illustrate the original vs. watermarked versions of both the LSB-based watermarking schemes for the BRISC and LIDC-IDRI datasets, respectively.

Results indicate that the watermarked images are nearly similar to the original images across both datasets. This can be confirmed by the reported PSNR values, which range from 60 to 62 dB in both datasets, indicating extremely high visual similarity between the original and the watermarked images. Moreover, the extracted watermarks have an NC value of 1 under no-attack conditions for all evaluated samples. However, even when visual quality is preserved, it cannot be assumed that watermarking has no impact on segmentation performance, nor can visual similarity alone reliably assess the robustness of segmentation outcomes. Thus, quantitative analysis is hence needed to determine its actual impact on the performance of segmentation.

#### 4.1.3. DWT-SVD Watermarking Results

**Figure 8:**
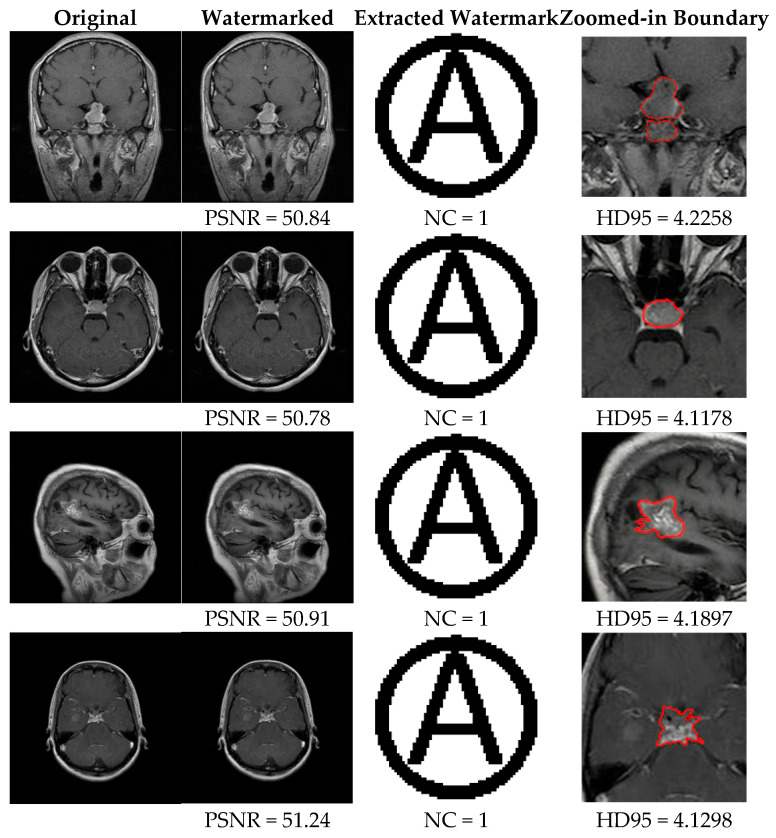
BRISC Dataset original vs. DWT-SVD-based watermarked samples and zoomed-in boundary overlay.

**Figure 9:**
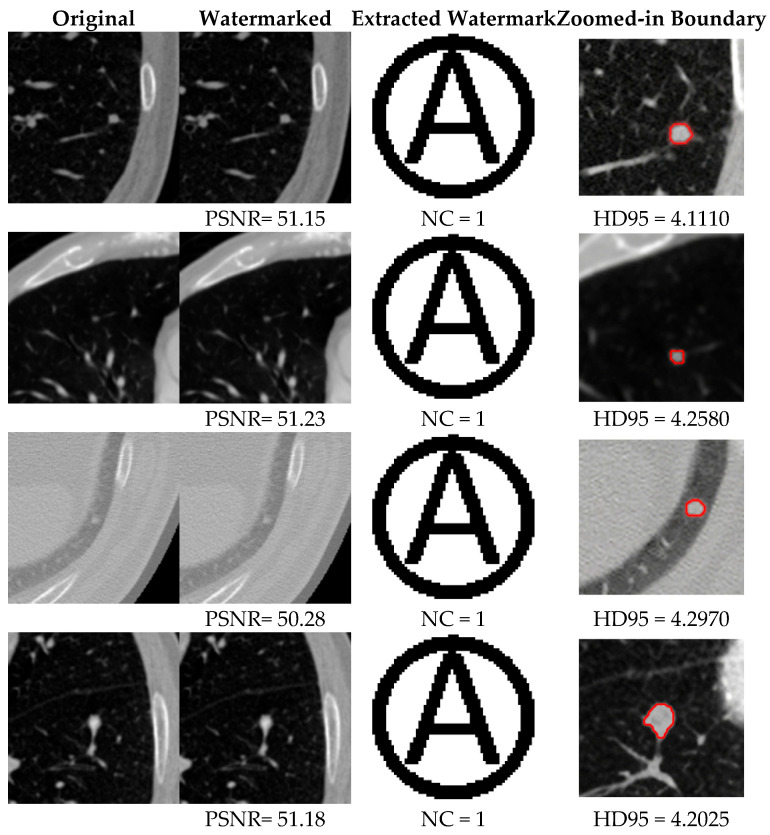
LIDC-IDRI Dataset original vs. DWT-SVD-based watermarked samples, images and zoomed-in boundary overlay visualisations.

Figure 8 and Figure 9 illustrate the original vs. watermarked versions of both the DWT-SVD-based watermarking schemes for BRISC and LIDC-IDRI datasets, respectively.

In the hybrid DWT-SVD scheme, PSNRs of watermarked images are almost the same in both datasets without attack, ranging from 50 to 51 dB, and NCs are almost the same at 1 without attack for all samples. However, the visual quality of preservation is not enough to conclude that the segmentation is not impacted; quantitative evaluation would be needed to assess the effect on segmentation performance.

### 4.2. Segmentation Results

To study the watermarking effect on segmentation tasks, both datasets must be trained and then evaluated using all targeted and proposed models with and without watermarking. Both datasets were split into 80% for training and validation and 20% for testing, following a widely adopted convention in deep learning workflows.

#### 4.2.1. BRISC Segmentation Results

The first experiment was conducted to evaluate the resistance of the segmentation models to the added watermark distortions on the BRISC samples. Each model was tested using clean data in addition to DWT, LSB, and hybrid DWT-SVD-based watermark samples. Table 4 shows the segmentation performance (mean ± standard deviation over 5 independent runs with different seeds) of all models on the BRISC dataset. In addition, the *p*-value metric is calculated based on the Wilcoxon signed-rank tests over the watermarked and no-watermarked conditions for the mIoU metric.

**Figure 10:**
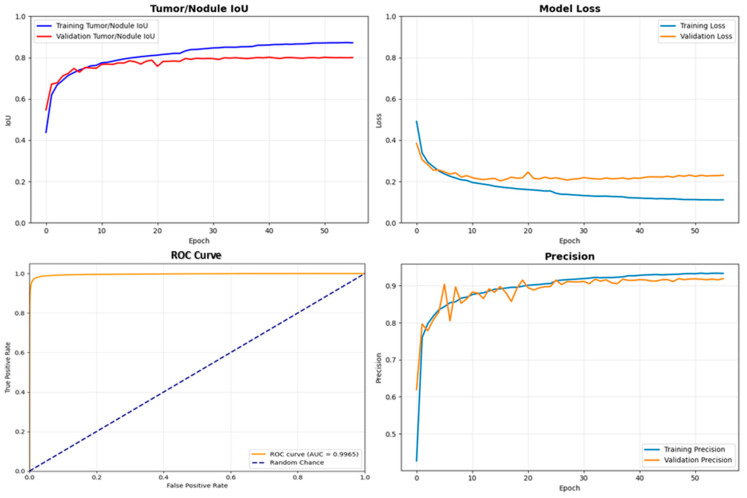
Training performance curves of the proposed AGFR-Net model on the BRISC dataset, including IoU, loss, ROC, and precision for both training and validation sets.

The results in Table 4 clearly indicate that watermarking leads to a very slight decrease in the performance across all targeted segmentation models. Specifically, taking the proposed model AGFR-Net as a benchmark, the DWT method leads to a reduction of approximately 0.44% in the mIoU and 0.1% in the DSC, while the LSB results in smaller decreases of about 0.37% and 0.02% for the same metrics. Similarly, the hybrid DWT-SVD watermarking scheme with (α=0.02) returns with the minimal reduction in the mIoU, with 0.145% and 0.011% for the DSC metric. This slight applies to all targeted segmentation models, including the proposed model. Additionally, statistical validation was performed using Wilcoxon signed-rank tests, resulting in *p*-values above 0.05, which indicates the observed degradations in performance are not statistically significant. Furthermore, it can be easily concluded that among the base models, both UNet and ResUNet++ consistently outperformed the established architectures due to their enhanced capabilities in extracting features from complex medical structures. However, the proposed AGFR-Net model specifically outperformed the baseline UNet model, achieving improvements of up to 1.53% in the mIoU metric and 2.23% in the DSC, along with a significant 19.8% reduction in HD95 score. These results demonstrate that adding multiple attention pathways provides considerable effects in producing more accurate and spatially consistent segmentation. However, while these statistics offer an outline of the final performance of the proposed model on the BRISC, they do not reflect the model’s behaviour during the training process. Consequently, it is necessary to provide an additional set of organised graphs that complement the tabular results by highlighting the overall training effectiveness, in addition to providing more in-depth knowledge of the learning dynamics in relation to the inputs provided. Figure 10 illustrates the training progress in terms of the IoU, loss, ROC, and precision curves.

**Figure 11:**
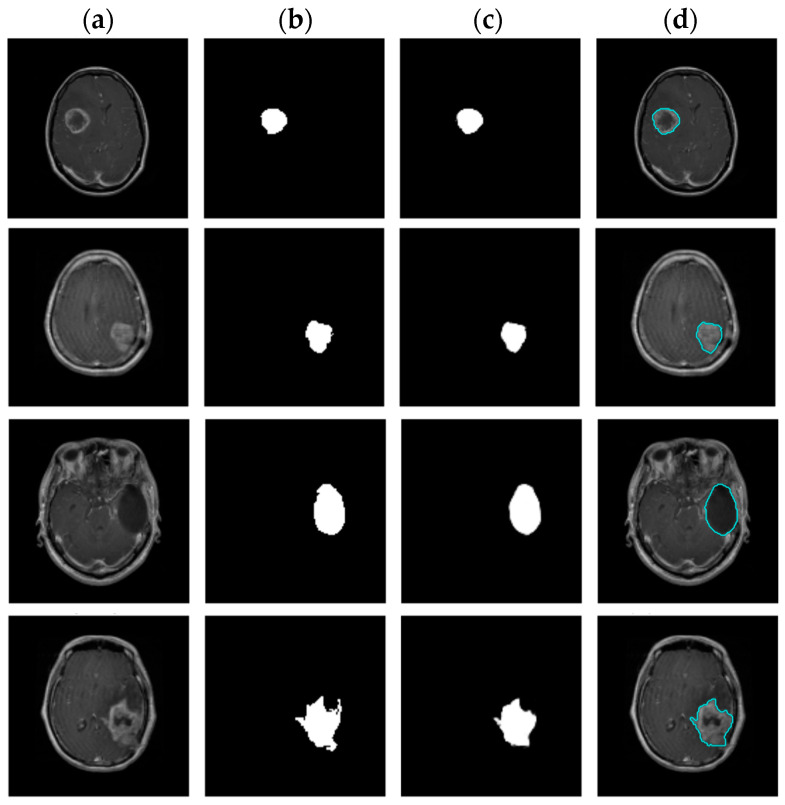
Segmentation results for a representative BRISC sample, showing (**a**) the original image, (**b**) the ground truth mask, (**c**) the predicted segmentation, and (**d**) the overlay of the predicted mask on the original image.

The presented curves show a stable learning process for the proposed model, with steady improvement across training cycles and close agreement between training and validation performance. Moreover, some qualitative segmentation samples from the BRISC dataset are illustrated in Figure 11. Each example includes the original image, ground truth mask, and predicted segmentation using the AGFR-Net. The results indicate a strong visual agreement between the predicted masks and the ground truth, with precise delineation of the target regions of interest of the brain.

#### 4.2.2. LIDC-IDRI Segmentation Results

Following the same framework, a second experiment was conducted, reapplying these processes to the LIDC-IDRI dataset as listed in Table 5.

Table 5 summarises the results of applying both watermarking techniques to the LIDC-IDRI dataset. In the DWT method, the decrease in the metrics remains limited, with the mIoU index decreasing by 0.29% and the DSC index by 0.39%. Similarly, the LSB method maintains all metrics within a narrow range, demonstrating exceptional stability, with an mIoU degradation of 0.22% and a DSC score of 0.19% over the proposed model. Furthermore, the hybrid DWT-SVD watermarking approach yields the lowest reduction in the mIoU and DSC metrics. These results reveal that similar trends can be observed: minor degradation in segmentation performance across all models, which confirms that watermarking has minimal impact on segmentation performance. Moreover, statistical validation was additionally carried out using Wilcoxon signed-rank tests, which produced *p*-values exceeding the threshold (0.05), revealing that the observed performance reductions are not statistically significant. Additionally, the results demonstrate that the proposed AGFR-Net model significantly outperforms all the baseline models in all evaluation metrics. Specifically, it achieved improvements of 3.51% in the mIoU score and 4.71% in the DSC score over the UNet model. This indicates enhanced accuracy in the pixel-level classification. In addition, the increased sensitivity score of 7.73% specifies the improved model’s ability to accurately detect targeted tumour regions. From another analytical perspective, the 16.52% decrease in the HD95 value indicates a significant enhancement in the segmentation accuracy of the proposed model and also confirms its resistance to distortions caused by watermarks, and it can effectively maintain segmentation quality under difficult conditions.

**Figure 12:**
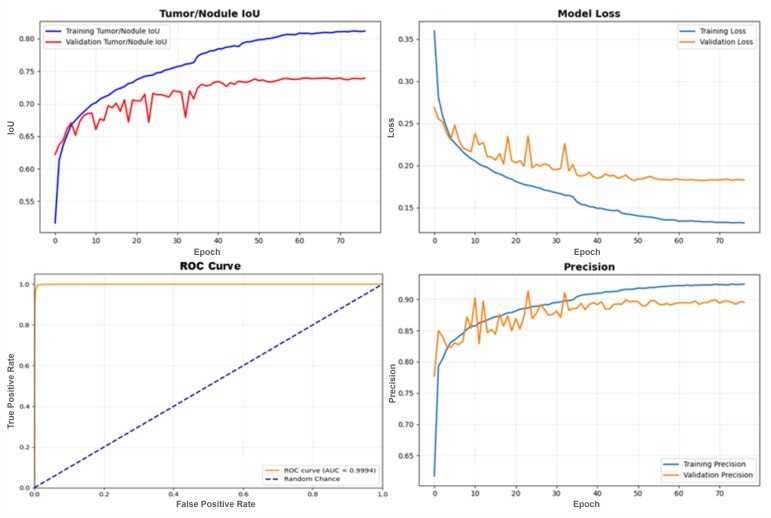
Training performance curves of the proposed AGFR-Net model on the LIDC-IDRI dataset, including IoU, loss, ROC, and precision for both training and validation sets.

Figure 12 displays the training behaviour and validation of the suggested model on the LIDC-IDRI dataset, which helps to improve the quantitative findings shown in Table 5.

**Figure 13:**
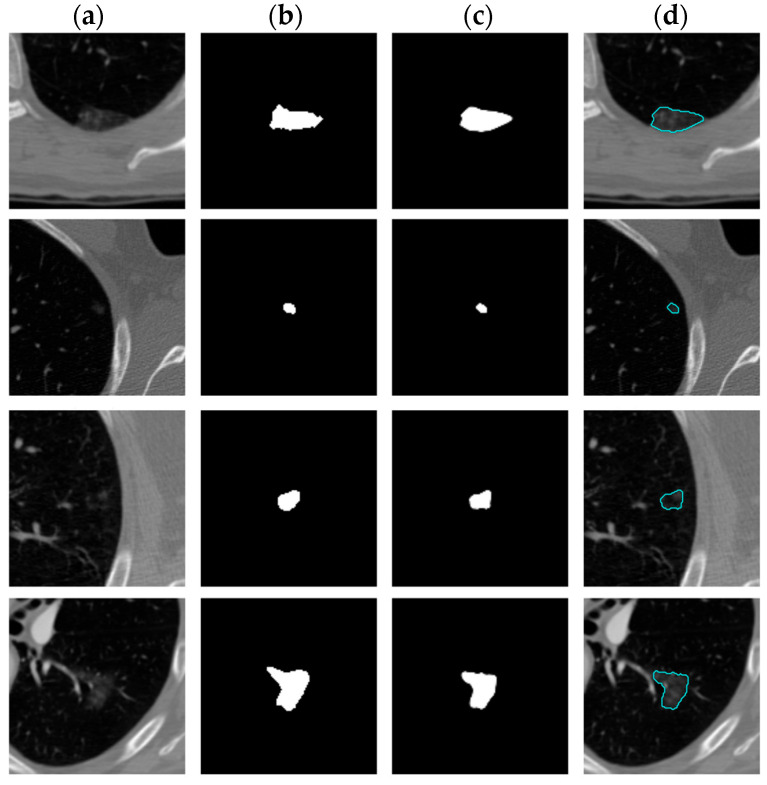
Segmentation results for a representative LIDC-IDRI sample, showing (**a**) the original image, (**b**) the ground truth mask, (**c**) the predicted segmentation, and (**d**) the overlay of the predicted mask on the original image.

The four presented subgraphs in Figure 12 reveal a stable learning approach, with effective alignment between training and validation performance. Furthermore, Figure 13 presents clarifying qualitative segmentation outcomes from the LIDC-IDRI dataset. Each sample consists of an overlay visual representation, the ground truth mask, the predicted segmentation, and the original samples. The results reveal a high degree of visual consistency between the predicted segmentations and the ground truth, with precise delineation of lung nodule regions of interest.

### 4.3. Effect of DWT Scaling Factors

This study also provides a deeper insight into the behaviour of watermarking under varying embedding strengths. Increasing the scaling factor leads to stronger watermark embedding, which introduces more noticeable modifications to pixel intensities, but it may also introduce distortions that negatively affect feature representation and segmentation accuracy. Therefore, to assess the impact of increasing the scaling factor on segmentation performance, a quantitative analysis was conducted. The findings offer insightful information on the trade-off between segmentation reliability and watermark resilience in medical imaging applications. Thus, the effect of the DWT embedding scaling factors was evaluated in depth at different scaling factors and consistently applied across all segmentation architectures to obtain clear and comprehensive analytical insights. As a first experiment, this approach was applied to the BRISC dataset, where three embedding scaling factors were defined, and their impact on segmentation performance was evaluated, as illustrated in Table 6.

The presented results clearly indicate that including watermarks leads to a very slight decrease in the performance of segmentation architectures, even at high embedding levels and across all segmentation models. Specifically, using the ResNet++ network as a benchmark for comparison and analysis, the analysis of different DWT scaling levels shows a minimal impact on segmentation performance. The lower the scaling factors, the lower the regression in the metrics. For instance, the best results are achieved at the $\left(\alpha_{1}=0.5,\alpha_{2}=1.5\right)$ scale, which provides the highest values for the mIoU and the DSC, as well as the lowest Hausdorff distance, indicating superior boundary conservation. These metrics regress slightly when the scaling factors are changed at the medium level, becoming more sensitive to feature distortion, particularly in edge and region representation.

This effect increases further at ($\alpha_{1}=1.5,\alpha_{2}=2$.5), resulting in a slight deterioration of up to 0.71% in sensitivity and a 1.94% increase in Hausdorff score compared to the scaling factors of ($\alpha_{1}=0.5,\alpha_{2}=1.5$). The results also show a slight degradation in segmentation performance over the proposed model AGFR-Net when there is a gradual increase in DWT embedding strength from the ($\alpha_{1}=0.5,\alpha_{2}=1.5$) to ($\alpha_{1}=1.5,\alpha_{2}=2.5$). Based on that, the specificity score decreased by up to 0.52%, and the Hausdorff score increased by 1.57%, indicating reduced boundary accuracy.

Another experiment was carried out using the same framework, by changing the type of the dataset to LIDC-IDRI while maintaining the same watermarking method. Accordingly, three embedding scaling factors were defined, and their impact on segmentation performance was evaluated. The presented results in Table 6 clearly indicate that including watermarks leads to a very slight decrease in the performance of segmentation architectures, even at high embedding scaling factors, and across all segmentation models. Specifically, using the ResNet++ network as a benchmark for comparison and analysis, the analysis of different DWT scaling levels shows a minimal impact on segmentation performance. The lower the scaling factors, the lower the regression in the metrics. For instance, the best results are achieved at the $\left(\alpha_{1}=0.5,\alpha_{2}=1.5\right)$ scale, which provides the highest values for the mIoU and the DSC, as well as the lowest Hausdorff distance, indicating superior boundary conservation. These metrics regress slightly when the scaling factors are changed at the medium level, becoming more sensitive to feature distortion, particularly in edge and region representation. This effect increases further at ($\alpha_{1}=1.5,\alpha_{2}=2.5$), resulting in a slight deterioration of up to 0.71% in sensitivity and a 1.94% increase in Hausdorff score compared to the scaling factors of ($\alpha_{1}=0.5,\alpha_{2}=1.5$). The results also show a slight degradation in segmentation performance over the proposed model AGFR-Net when there is a gradual increase in DWT embedding strength from the ($\alpha_{1}=0.5,\alpha_{2}=1.5$) to ($\alpha_{1}=1.5,\alpha_{2}=2.5$). Based on that, the specificity score decreased by up to 0.52%, and the Hausdorff score increased by 1.57%, indicating reduced boundary accuracy.

Following the same approach, an additional experiment was conducted, changing the type of the dataset to LIDC-IDRI while maintaining the same watermarking method. Accordingly, three embedding scaling factors were defined, and their impact on segmentation performance was evaluated, as illustrated in Table 7.

Table 7 summarises the results of applying the DWT watermarking technique to the LIDC-IDRI dataset. Similar trends can be observed, with performance declining only slightly as watermark density increases. However, all models maintain their high performance, especially the proposed model. In more detail, taking the ResNet++ model as a reference, the listed results show a consistent and gradual reduction in segmentation performance as the scaling levels of the DWT increase. Specifically, when the embedding level increases from the ($\alpha_{1}=0.5,\alpha_{2}=1.5$) to ($\alpha_{1}=1.5,\alpha_{2}=2.5$), the DSC score decreased by 0.22%, whereas the specificity and sensitivity decreased by 0.23%. These scores are accomplished with a slight increase within the Hausdorff metric, an increase that indicates a slight deterioration in boundary accuracy. Similarly, the proposed model AGFR-Net was also affected in the same manner, with a gradual degradation in the pixel-level performance as the DWT embedding scaling factor increases from the ($\alpha_{1}=0.5,\alpha_{2}=1.5$) to ($\alpha_{1}=1.5,\alpha_{2}=2.5$). Specifically, the mIoU score decreased by 0.15%, while the specificity and sensitivity metrics decreased by up to 0.31% and 0.24%, respectively.

It can be concluded here that despite the high sensitivity of the proposed model, which is supported by the inclusion of the attention units, the effect of watermark inclusion and resulting distortions remains minimal. More specifically, increasing the embedding scaling factor on medical images in DWT was systematically tested, and all attempts showed a very limited negative impact on segmentation performance. On the other hand, the proposed model AGFR-Net consistently outperforms the benchmarked models under all conditions, demonstrating its high robustness, even though its performance on the LIDC-IDRI dataset is generally higher than on the BRISC dataset due to the increased variability and complexity of brain tissue. Based on the analysis, three key findings can be drawn. First, embedding watermarks using different techniques and strengths has only a minimal impact on segmentation performance across different datasets. Second, the proposed attention-enhanced segmentation model demonstrates high efficiency and robustness, making it a strong candidate for real-world medical image analysis applications. Third, the numerical difference in the observed performances may have different clinical significance for different applications downstream (such as tumour volume estimation and radiotherapy planning). However, the accuracy of segmentations and the consistency of the boundaries do not show systematic degradation.

### 4.4. Ablation Study of AGFR-Net

To determine the individual contribution of each of the added enhancement components to the AGFR architecture, a systematic ablation study was conducted using identical settings on both databases. This study targeted all the attention modules, in addition to the proposed boundary improvement module, as shown in Table 8.

The ablation results show that the performance of each added component is beneficial for segmentation. More specifically, the RDA brings the maximum improvement, followed by AG and BR, which indicates that feature refinement and attention mechanisms play a stronger role than boundary refinement alone. Furthermore, the combination of these modules results in extra gains, especially for the RDA + AG combination. The final configuration that forms the AGFR-Net is the best in all the measures over the baseline and intermediate variants for both datasets. This gain is accompanied by a moderate increase in model complexity and a reasonable trade-off between the segmentation performance and computational cost.

## 5. Conclusions

This article examined how digital watermarking influences the performance of medical image segmentation using deep learning. Unlike prior work focused on watermark imperceptibility, robustness, or classification-based analysis, this study evaluates the impact of watermarking on segmentation performance at the pixel level. To this end, three representative watermarking schemes, spatial-domain LSB, frequency-domain DWT, and hybrid DWT-SVD, which combines wavelet analysis with singular value decomposition, were applied to the BRISC and LIDC-IDRI datasets, and their impact on segmentation performance was evaluated across five well-established architectures, in addition to the proposed lightweight AGFR-Net model. The experimental findings revealed that the watermarking process leads to minimal degradation in segmentation performance across both datasets and all evaluated models. On the BRISC dataset, the DWT results in a slight decrease of 0.44% in mIoU and 0.10% in DSC, while LSB shows smaller reductions of 0.37% and 0.02%, respectively. Similarly, the hybrid DWT-SVD scheme returns the smallest reduction with 0.145% in mIoU and 0.011% in DSC metric. This slight decrease also applies to the LIDC-IDRI dataset across both watermarking methods. These outcomes validate that the net impact of watermarking on segmentation performance is marginal and can be negligible compared to the non-watermarked baseline.

Moreover, the proposed AGFR-Net model was the most successful in terms of overall performance and robustness compared to the other analysed architectures. It implies that feature refinement based on attention can enhance the accuracy of segmentation without losing resistance to imperceptible watermark-induced perturbations. These findings demonstrate the feasibility of integrating watermark-based protection into AI-driven medical image segmentation pipelines. Overall, the study provides quantitative evidence that, when carefully implemented, watermarking introduces minimal impact on segmentation performance. These findings highlight the effectiveness of attention-based, biologically inspired segmentation frameworks, which maintain robust diagnostic performance even in the presence of watermarks.

This work, however, has several limitations that can be addressed in future studies. First, this study relies on two datasets; therefore, further validation across a broader range of modalities, such as retinal imaging and histopathology, is needed to ensure generalisability across a wider array of clinical applications. Second, subsequent research could also investigate additional watermarking techniques such as deep-learning-based watermarking schemes, adaptive watermarking, and reversible watermarking and consider various embedding strengths and payload capacities. Third, the evaluation of segmentation performance could be extended to recent architectures, such as the TransUNet, Swin-UNet, and nnU-Net, to identify whether the determined robustness trends are still present in modern segmentation paradigms. Fourth, more realistic deployment settings, such as varying payloads, embedding strengths, compression, noise, geometric distortions, and hybrid degradation scenarios, should be explored in greater detail. Finally, future investigations could include further relevant clinical analyses, such as the boundary consistency analysis, small lesion sensitivity, and evaluation of downstream clinical impact, such as tumour volume estimation and treatment planning sensitivity.

## Figures and Tables

**Table 1:** Architectural summary of the five baseline segmentation models.

Model	Architecture Type	Encoder/Backbone	Skip Connections	Key Feature	Params (M)	FLOPs (G)	Inference Time (ms)	FPS
U-Net	CNN Encoder–Decoder	Custom CNN	Applied	Multi-scale skip fusion.	31.03	90.03	59.58	16.78
ResUNet++	Residual CNN Encoder- Decoder	Residual CNN encoder	Attention gated	Residual learning with attention mechanisms.	34.49	9.24	48.32	20.70
SegNet	CNN Encoder–Decoder	Custom CNN	Not applied (uses pooling indices)	Transposed conv upsampling.	29.46	80.10	61.33	16.30
FCDenseNet	Dense Encoder–Decoder	DenseNet based encoder	Partially Applied	Dense connectivity, efficient.	14.59	12.91	75.13	13.31
TernausNet	CNN-based U-Net	VGG-11 Pretrained encoder	Applied	Transfer learning with VGG-based encoder	22.93	46.44	58.89	16.98

**Table 2:** Architectural summary of the proposed segmentation model.

Model	Architecture Type	Encoder/ Backbone	Skip Connections	Key Feature	Params (M)	FLOPs (G)	Inference Time (ms)	FPS
AGFR-Net	CNN Encoder– Decoder with attention	Custom CNN with dilated Convolution	Applied with attention gates	Lightweight; channel and spatial attentions, and multiscale capability	9.68	1.689	20.80	48.1

**Table 3:** Training hyperparameters.

Training Option	Value
Optimizer	Adam (β_1_ = 0.9, β_2_ = 0.999, ε = 1 × 10^−7^)
Initial learning rate	0.0001
Learning rate drop factor	0.5 (ReduceLROnPlateau, patience = 5, min_lr = 1 × 10^−6^)
Max epochs	100
Shuffle	Every epoch
Validation frequency	Every epoch
Execution environment	Kaggle GPU (Mirrored Strategy)
Mini-batch size	4
Loss function	Loss = 0.6 × FocalTversky + 0.4 × Weighted BCE
Input image size (H × W)	128 × 128 (resized from 256 × 256)
Early stopping—patience	15 epochs

**Table 4:** Performance evaluation of segmentation models on the BRISC dataset using the DWT, LSB, and hybrid DWT-SVD watermarking methods.

Model	Test Set Condition	mIoU	DSC	Specificity	Sensitivity	HD95	*p*-Value
UNet	Original	0.8741 _±0.0022_	0.8591 _±0.0024_	0.8471 _±0.0026_	0.8713 _±0.0029_	5.2031 _±0.11_	–
	DWT	0.8718 _±0.0025_	0.8562 _±0.0026_	0.8464 _±0.0028_	0.8667 _±0.0031_	5.2522 _±0.13_	0.082
	LSB	0.8722 _±0.0023_	0.8567 _±0.0025_	0.8394 _±0.0027_	0.8752 _±0.0030_	5.2251 _±0.12_	0.120
	DWT-SVD	0.8733 _±0.0026_	0.8576 _±0.0027_	0.8467 _±0.0029_	0.8701 _±0.0032_	5.2125 _±0.14_	0.132
ResUNet++	Original	0.8704 _±0.0021_	0.8609 _±0.0023_	0.8431 _±0.0025_	0.8773 _±0.0027_	5.1096 _±0.10_	–
	DWT	0.8675 _±0.0024_	0.8558 _±0.0026_	0.8340 _±0.0028_	0.8692 _±0.0030_	5.1981 _±0.12_	0.091
	LSB	0.8683 _±0.0023_	0.8569 _±0.0025_	0.8405 _±0.0027_	0.8652 _±0.0029_	5.1771 _±0.11_	0.140
	DWT-SVD	0.8696 _±0.0025_	0.8587 _±0.0025_	0.8418 _±0.0029_	0.8721 _±0.0038_	5.1451 _±0.14_	0.155
**SegNet**	Original	0.8557 _±0.0026_	0.8353 _±0.0028_	0.8215 _±0.0031_	0.8495 _±0.0033_	6.0094 _±0.15_	–
	DWT	0.8539 _±0.0029_	0.8329 _±0.0031_	0.8181 _±0.0034_	0.8405 _±0.0036_	6.1185 _±0.16_	0.111
	LSB	0.8546 _±0.0028_	0.8339 _±0.0030_	0.8298 _±0.0033_	0.8459 _±0.0035_	6.1025 _±0.15_	0.190
	DWT-SVD	0.8544 _±0.0033_	0.8340 _±0.0032_	0.8285 _±0.0035_	0.8435 _±0.0032_	6.1004 _±0.20_	0.216
FCDenseNet	Original	0.8353 _±0.0031_	0.8078 _±0.0033_	0.7786 _±0.0036_	0.8854 _±0.0031_	6.2258 _±0.17_	–
	DWT	0.8323 _±0.0034_	0.8042 _±0.0036_	0.7721 _±0.0039_	0.8757 _±0.0034_	6.4958 _±0.19_	0.152
	LSB	0.8349 _±0.0032_	0.8052 _±0.0034_	0.7722 _±0.0037_	0.8775 _±0.0033_	6.4458 _±0.18_	0.481
	DWT-SVD	0.8350 _±0.0031_	0.8068 _±0.0035_	0.7756 _±0.0036_	0.8784 _±0.0035_	6.3685 _±0.27_	0.490
TernausNet	Original	0.8651 _±0.0024_	0.8476 _±0.0026_	0.8443 _±0.0029_	0.8581 _±0.0031_	5.4011 _±0.13_	–
	DWT	0.8630 _±0.0027_	0.8441 _±0.0029_	0.8392 _±0.0032_	0.8531 _±0.0034_	5.5412 _±0.15_	0.110
	LSB	0.8645 _±0.0025_	0.8469 _±0.0027_	0.8441 _±0.0030_	0.8547 _±0.0032_	5.4412 _±0.14_	0.181
	DWT-SVD	0.8648 _±0.0030_	0.8472 _±0.0032_	0.8442 _±0.0038_	0.8558 _±0.0022_	5.4216 _±0.20_	0.197
AGFR-Net	Original	0.8888 _±0.0019_	0.8761 _±0.0020_	0.9029 _±0.0021_	0.8838 _±0.0023_	4.1551 _±0.09_	–
	DWT	0.8849 _±0.0021_	0.8752 _±0.0022_	0.8992 _±0.0024_	0.8827 _±0.0025_	4.2082 _±0.11_	0.121
	LSB	0.8855 _±0.0020_	0.8757 _±0.0021_	0.9016 _±0.0023_	0.8833 _±0.0024_	4.1915 _±0.10_	0.136
	DWT-SVD	0.8875 _±0.0030_	0.8760 _±0.0033_	0.9021 _±0.0038_	0.8836 _±0.0025_	4.1758 _±0.18_	0.141

**Table 5:** Performance evaluation of segmentation models on the LIDC-IDRI dataset using the DWT, LSB, and hybrid DWT-SVD watermarking methods.

Model	Test Set Condition	mIoU	DSC	Specificity	Sensitivity	HD95	*p*-Value
UNet	Original	0.8789 _±0.0033_	0.8607 _±0.0032_	0.9069 _±0.0030_	0.8341 _±0.0022_	4.8031 _±0.0028_	–
	DWT	0.8728 _±0.0028_	0.8568 _±0.0030_	0.8821 _±0.0029_	0.8274 _±0.0028_	4.9024 _±0.0030_	0.077
	LSB	0.8747 _±0.0024_	0.8582 _±0.0028_	0.8851 _±0.0025_	0.8307 _±0.0025_	4.8053 _±0.0023_	0.111
	DWT-SVD	0.8764 _±0.0029_	0.8597 _±0.0033_	0.8901 _±0.0022_	0.8322 _±0.0027_	4.8044 _±0.0031_	0.129
ResUNet++	Original	0.8843 _±0.0022_	0.8705 _±0.0028_	0.8941 _±0.0028_	0.8670 _±0.0025_	4.6091 _±0.0030_	–
	DWT	0.8796 _±0.0025_	0.8644 _±0.0022_	0.8852 _±0.0029_	0.8557 _±0.0028_	4.6411 _±0.0028_	0.081
	LSB	0.8818 _±0.0033_	0.8672 _±0.0025_	0.8880 _±0.0033_	0.8566 _±0.0033_	4.6123 _±0.0025_	0.136
	DWT-SVD	0.8820 _±0.0035_	0.8679 _±0.0023_	0.8900 _±0.0037_	0.8606 _±0.0032_	4.6027 _±0.0036_	0.142
**SegNet**	Original	0.8694 _±0.0032_	0.8445 _±0.0030_	0.8804 _±0.0024_	0.8075 _±0.0027_	5.3981 _±0.0025_	–
	DWT	0.8609 _±0.0025_	0.8400 _±0.0024_	0.8663 _±0.0023_	0.8054 _±0.0025_	5.4421 _±0.0039_	0.113
	LSB	0.8616 _±0.0038_	0.8410 _±0.0022_	0.8728 _±0.0028_	0.8014 _±0.0033_	5.4401 _±0.0040_	0.180
	DWT-SVD	0.8637 _±0.0031_	0.8431 _±0.0027_	0.8784 _±0.0030_	0.8044 _±0.0038_	5.4203 _±0.0026_	0.224
FCDenseNet	Original	0.8721 _±0.0035_	0.8578 _±0.0028_	0.9056 _±0.0027_	0.8006 _±0.0028_	4.7731 _±0.0024_	–
	DWT	0.8645 _±0.0025_	0.8448 _±0.0026_	0.8946 _±0.0028_	0.7974 _±0.0028_	4.8044 _±0.0032_	0.172
	LSB	0.8655 _±0.0027_	0.8471 _±0.0036_	0.9021 _±0.0035_	0.7998 _±0.0028_	4.8084 _±0.0035_	0.387
	DWT-SVD	0.8705 _±0.0030_	0.8521 _±0.0034_	0.9037 _±0.0025_	0.8000 _±0.0022_	4.7981 _±0.0036_	0.368
TernausNet	Original	0.8746 _±0.0026_	0.8585 _±0.0031_	0.8573 _±0.0028_	0.8668 _±0.0022_	4.8231 _±0.0036_	–
	DWT	0.8719 _±0.0021_	0.8542 _±0.0025_	0.8551 _±0.0022_	0.8590 _±0.0038_	5.0144 _±0.0035_	0.190
	LSB	0.8738 _±0.0027_	0.8577 _±0.0024_	0.8562 _±0.0036_	0.8594 _±0.0027_	4.8143 _±0.0024_	0.251
	DWT-SVD	0.8739 _±0.0022_	0.8580 _±0.0027_	0.8568 _±0.0031_	0.8604 _±0.0021_	4.8193 _±0.0021_	0.300
AGFR-Net	Original	0.9075 _±0.0021_	0.9004 _±0.0025_	0.9081 _±0.0028_	0.8973 _±0.0029_	3.8891 _±0.0027_	–
	DWT	0.9049 _±0.0023_	0.8969 _±0.0029_	0.8992 _±0.0028_	0.8937 _±0.0026_	4.0181 _±0.0033_	0.127
	LSB	0.9055 _±0.0032_	0.8987 _±0.0038_	0.9072 _±0.0028_	0.8949 _±0.0031_	4.0117 _±0.0024_	0.131
	DWT-SVD	0.9058 _±0.0029_	0.9000 _±0.0035_	0.9080 _±0.0021_	0.8950 _±0.0027_	3.9178 _±0.0020_	0.149

**Table 6:** Performance evaluation of segmentation models on the BRISC dataset using the DWT watermarking method at different levels.

Model	Scaling Factor	mIoU	DSC	Spec.	Sens.	HD95
UNet	$\alpha_{1}=0.5,\alpha_{2}=1.5$	0.8698	0.8537	0.8381	0.8701	5.2723
	$\alpha_{1}=1,\alpha_{2}=2$	0.8691	0.8532	0.8378	0.8692	5.2915
	$\alpha_{1}=1.5,\alpha_{2}=2.5$	0.8687	0.8526	0.8359	0.8659	5.3001
ResUNet++	$\alpha_{1}=0.5,\alpha_{2}=1.5$	0.8647	0.8531	0.8323	0.8675	5.2001
	$\alpha_{1}=1,\alpha_{2}=2$	0.8640	0.8520	0.8315	0.8643	5.2291
	$\alpha_{1}=1.5,\alpha_{2}=2.5$	0.8635	0.8502	0.8310	0.8614	5.3012
SegNet	$\alpha_{1}=0.5,\alpha_{2}=1.5$	0.8532	0.8320	0.8161	0.8485	6.2117
	$\alpha_{1}=1,\alpha_{2}=2$	0.8529	0.8319	0.8158	0.8481	6.1901
	$\alpha_{1}=1.5,\alpha_{2}=2.5$	0.8522	0.8310	0.8151	0.8477	6.1899
FCDenseNet	$\alpha_{1}=0.5,\alpha_{2}=1.5$	0.8251	0.8030	0.7701	0.8725	6.5015
	$\alpha_{1}=1,\alpha_{2}=2$	0.8234	0.8017	0.7692	0.8716	6.5119
	$\alpha_{1}=1.5,\alpha_{2}=2.5$	0.8228	0.8002	0.7687	0.8705	6.5124
TernausNet	$\alpha_{1}=0.5,\alpha_{2}=1.5$	0.8626	0.8439	0.8388	0.8522	5.5513
	$\alpha_{1}=1,\alpha_{2}=2$	0.8620	0.8435	0.8375	0.8512	5.4521
	$\alpha_{1}=1.5,\alpha_{2}=2.5$	0.8615	0.8430	0.8366	0.8502	5.4601
AGFR-Net	$\alpha_{1}=0.5,\alpha_{2}=1.5$	0.8842	0.8743	0.8959	0.8818	4.2108
	$\alpha_{1}=1,\alpha_{2}=2$	0.8839	0.8738	0.8928	0.8805	4.2414
	$\alpha_{1}=1.5,\alpha_{2}=2.5$	0.8832	0.8730	0.8912	0.8800	4.2771

**Table 7:** Performance evaluation of segmentation models on the LIDC-IDRI dataset using the DWT watermarking method at different levels.

Model	WaterMarking	mIoU	DSC	Spec.	Sens.	HD95
UNet	$\alpha_{1}=0.5,\alpha_{2}=1.5$	0.8717	0.8560	0.8819	0.8268	4.9064
	$\alpha_{1}=1,\alpha_{2}=2$	0.8710	0.8549	0.8810	0.8264	4.9107
	$\alpha_{1}=1.5,\alpha_{2}=2.5$	0.8701	0.8537	0.8802	0.8259	4.9124
ResUNet++	$\alpha_{1}=0.5,\alpha_{2}=1.5$	0.8786	0.8637	0.8849	0.8547	4.6851
	$\alpha_{1}=1,\alpha_{2}=2$	0.8782	0.8629	0.8835	0.8534	4.6914
	$\alpha_{1}=1.5,\alpha_{2}=2.5$	0.8779	0.8618	0.8829	0.8527	4.6988
SegNet	$\alpha_{1}=0.5,\alpha_{2}=1.5$	0.8598	0.8392	0.8646	0.8014	5.4701
	$\alpha_{1}=1,\alpha_{2}=2$	0.8581	0.8380	0.8621	0.8003	5.4881
	$\alpha_{1}=1.5,\alpha_{2}=2.5$	0.8570	0.8369	0.8607	0.7998	5.4981
FCDenseNet	$\alpha_{1}=0.5,\alpha_{2}=1.5$	0.8633	0.8438	0.8939	0.7965	4.8262
	$\alpha_{1}=1,\alpha_{2}=2$	0.8625	0.8421	0.8925	0.7957	4.8311
	$\alpha_{1}=1.5,\alpha_{2}=2.5$	0.8613	0.8419	0.8913	0.7946	4.8424
TernausNet	$\alpha_{1}=0.5,\alpha_{2}=1.5$	0.8714	0.8536	0.8546	0.8577	5.0162
	$\alpha_{1}=1,\alpha_{2}=2$	0.8702	0.8528	0.8532	0.8555	5.1121
	$\alpha_{1}=1.5,\alpha_{2}=2.5$	0.8695	0.8518	0.8528	0.8548	5.1284
AGFR-Net	$\alpha_{1}=0.5,\alpha_{2}=1.5$	0.9040	0.8943	0.8979	0.8928	4.0262
	$\alpha_{1}=1,\alpha_{2}=2$	0.9033	0.8934	0.8968	0.8919	4.0221
	$\alpha_{1}=1.5,\alpha_{2}=2.5$	0.9026	0.8928	0.8952	0.8907	4.0303

**Table 8:** Ablation study of AGFR-Net architecture on BRSIC and LIDC-IDRI datasets.

Model Variant	Params (M)	BRISC			LIDC-IDRI		
		mIoU	DSC	HD95	mIoU	DSC	HD95
Baseline	6.86	0.8766	0.8631	4.6501	0.8961	0.8886	4.3500
Baseline + RDA	7.81	0.8841 _(+0.86%)_	0.8721 _(+1.04%)_	4.3801 _(−5.81%)_	0.9046 _(+0.95%)_	0.8981 _(+1.07%)_	4.0500 _(−6.90%)_
Baseline + AG	7.61	0.8816 _(+0.57%)_	0.8691 _(+0.70%)_	4.4201 _(−4.95%)_	0.9016 _(+0.61%)_	0.8946 _(+0.68%)_	4.1000 _(−5.75%)_
Baseline + BR	7.26	0.8796 _(+0.34%)_	0.8661 _(+0.35%)_	4.4800 _(−3.66%)_	0.8996 _(+0.39%)_	0.8921 _(+0.39%)_	4.2002 _(−3.45%)_
Baseline + RDA + AG	8.56	0.8866 _(+1.14%)_	0.8746 _(+1.33%)_	4.2202 _(−9.25%)_	0.9061 _(+1.12%)_	0.8996 _(+1.24%)_	3.9600 _(−8.97%)_
Baseline + RDA + BR	8.21	0.8851 _(+0.97%)_	0.8731 _(+1.16%)_	4.2800 _(−7.96%)_	0.9051 _(+1.00%)_	0.8981 _(+1.07%)_	4.0000 _(−8.05%)_
Baseline + AG + BR	8.01	0.8831 _(+0.74%)_	0.8706 _(+0.87%)_	4.3501 _(−6.45%)_	0.9031 _(+0.78%)_	0.8961 _(+0.84%)_	4.0801 _(−6.21%)_
AGFR-Net	9.68	0.8888 _(+1.40%)_	0.8761 _(+1.52%)_	4.1551 _(−10.64%)_	0.9075 _(+1.28%)_	0.9004 _(+1.34%)_	3.8891 _(−10.60%)_

## Data Availability

Datasets can be downloaded from: https://www.kaggle.com/datasets/briscdataset/brisc2025 (accessed on 7 February 2026) and https://www.cancerimagingarchive.net/collection/lidc-idri/ (accessed on 10 February 2026).
